# Plasma Chemerin Levels Are Increased in ST Elevation Myocardial Infarction Patients with High Thrombus Burden

**DOI:** 10.1155/2018/5812704

**Published:** 2018-03-26

**Authors:** Ahmet Hakan Ateş, Uğur Arslan, Aytekin Aksakal, Ahmet Yanık, Metin Özdemir, Selim Kul

**Affiliations:** ^1^Department of Cardiology, Samsun Training and Research Hospital, University of Health Sciences, Samsun, Turkey; ^2^Department of Microbiology, Gazi State Hospital, Samsun, Turkey; ^3^Department of Cardiology, Ahi Evren Training and Research Hospital, University of Health Sciences, Trabzon, Turkey

## Abstract

**Objective:**

To investigate plasma chemerin levels in ST elevation myocardial infarction (STEMI) patients and find out possible relationships between plasma chemerin levels and angiographic characteristics.

**Patients and Methods:**

Ninety-seven consecutive patients who presented with STEMI and underwent primary percutaneous coronary intervention (PCI) with coronary stents were enrolled, and 30 age- and sex-matched patients with stable angina pectoris who underwent coronary angiography formed the control group. Angiographic characteristics of the patients including thrombolysis in myocardial infarction (TIMI) thrombus and Gensini scores were noted. Blood samples were taken to detect several biochemical markers including plasma chemerin levels at the admission to hospital.

**Results:**

Serum chemerin and C-reactive protein (CRP) levels were significantly increased in patients with STEMI. Among STEMI patients, serum chemerin levels were significantly higher in patients with high thrombus burden (581.5 ± 173.7 versus 451.3 ± 101.2 mg/dL, *p* < 0.001). CRP levels and peak creatine kinase-MB (CK-MB) levels were higher, and left ventricular ejection fraction and post-PCI TIMI flow were lower in patients with high thrombus burden. After multivariate analysis, serum chemerin levels were also higher in patients with high thrombus grade (odds ratio: 1.009 (1.005–1.014), *p* < 0.001). Besides, serum chemerin levels were also found to be significantly correlated with CRP (*r*=0.47, *p* < 0.001) and peak CK-MB (*r*=0.376, *p* < 0.001) levels.

**Conclusions:**

Results from our study have demonstrated for the first time that chemerin levels were higher in STEMI patients with greater thrombus burden and higher level of inflammation.

## 1. Introduction

Acute ST segment elevation myocardial infarction (STEMI) most commonly occurs when thrombus formation results in complete occlusion of a major epicardial coronary artery. The most serious form of acute coronary syndromes, STEMI is a life-threatening situation, and it must be diagnosed and treated promptly via coronary revascularization, usually by percutaneous coronary intervention or fibrinolytic therapy. Several biomarkers have been studied in STEMI to predict the prognosis of this disease which mainly develops due to an atherosclerotic and thrombotic condition.

Adipose tissue not only serves as a tissue of fat for energy storage, but also behaves like an active endocrine organ secreting cytokines and bioactive adipokines, such as leptin, adiponectin, tumor necrosis factor-alpha (TNF-*α*), and resistin [1–3]. Adipokines have several systemic effects on different organs such as liver, brain, vasculature, and endothelium. They have an effect on endothelial cells, arterial smooth muscle cells, and macrophages all of which have different roles in the atherosclerotic process [4].

Chemerin, also known as tazarotene-induced gene 2 protein (TIG2) or retinoic acid receptor responder 2 (RARRES2), is a recently discovered adipocytokine that is released from liver and white fat tissue. It has been shown to regulate adipocyte differentiation, maturation, and metabolism [5, 6], and it modulates the expression of adipocyte genes, such as glucose transporter-4, adiponectin, and leptin. These genes are involved in lipid homeostasis [7, 8]. Several studies have reported that chemerin levels are also associated with inflammation, adipogenesis, components of metabolic syndromes, lipid homeostasis, atherosclerosis, and peripheral arterial stiffness [9–11]. Circulating chemerin levels are found to be elevated in multiple metabolic and inflammatory diseases such as type 2 diabetes, metabolic syndrome, psoriasis, and cardiovascular diseases [12]. The role of chemerin in pathogenesis and development of cardiovascular disease and atherosclerosis has been investigated in some trials, and a positive correlation between chemerin secretion and coronary atherosclerosis has been shown by Spiroglou et al. [13]. However, negative studies are also present.

Herein, we studied the circulating chemerin levels in patients with STEMI undergoing primary percutaneous coronary intervention (PCI). Our aim was to assess the relationship between chemerin levels and several clinical and angiographic parameters, especially the thrombus burden in patients with STEMI.

## 2. Methods

### 2.1. Study Population

We enrolled 97 consecutive patients who presented with STEMI and underwent primary PCI with coronary stents at the Samsun Training and Research Hospital between January 2016 and January 2017. These patients were presented with STEMI within the first 12 hours after the onset of pain and had an ST segment elevation at least 1 mm (2 mm for V1-V3) in two or more contiguous leads in electrocardiogram (ECG) or new onset left bundle-branch block. Patients with cardiogenic shock, admission 12 h after the onset of chest pain, history of renal dysfunction (serum creatinine ≥ 1.5 mg/dL), evidence of malignant disease, or unwillingness to participate were excluded from the study. Control group was constituted from 30 age- and sex-matched patients with stable angina pectoris who underwent coronary angiography. Stable angina pectoris was defined as stable anginal or equivalent symptoms lasting for at least one month.

The characteristics of patients and medical history such as age, sex, history of hypertension, diabetes, hyperlipidemia, and smoking status were recorded. Additionally, serum creatinine, lipid panel, cardiac biomarkers such as creatine kinase-MB (CK-MB), and C-reactive protein (CRP) were measured for all patients during hospital admission. Blood samples for chemerin were taken just before percutaneous coronary intervention at the admission to hospital.

Human Chemerin (CHEMERIN) ELISA kit (Hangzhou Eastbiopharm Co. Hangzhou, China), Synergy 4 Microplate Reader (BioTek, USA), and a Multiwash (TriContinent Scientific, USA) microplate washer were used to measure serum chemerin levels (ng/mL) which were calculated according to a standard curve which was formed using a curvetting equation.

### 2.2. Clinical and Angiographic Definitions

Hypertension (HTN) was defined as systolic pressure >140 mm Hg and/or a diastolic pressure >90 mm Hg or if the patient was taking antihypertensive medications before the admission to our center. The diagnosis of diabetes mellitus (DM) was defined as a previous history of DM treated with drug therapies or patients with HbA1c concentrations over 6.5% at admission. Hyperlipidemia was defined as total cholesterol level >200 mg/dL or previous history of statin use. Current smokers were defined as those who had smoked for some period during the past year. Left ventricular ejection fraction (LVEF) was measured using the modified Simpson's method with transthoracic echocardiography, which was performed on the third day of STEMI.

Coronary angioplasty and stent implantation were performed according to standard percutaneous techniques through the femoral artery. Standard selective coronary angiography with at least 4 views of the left coronary system and 2 views of the right coronary artery was performed using the Judgkins technique. All the patients who were admitted with acute STEMI underwent emergent PCI to the culprit vessel with drug-eluting stents.

The Gensini score considering the extent and severity of the lesions at coronary angiography was calculated for each patient [14]. Coronary artery stenosis was measured visually and scored as 1 for 1–25% stenosis, 2 for 26–50% stenosis, 4 for 51–75% stenosis, 8 for 76–90% stenosis, 16 for 91–99% stenosis, and 32 for total occlusion. Then, this score was multiplied by a constant number which was obtained according to the anatomical position of the lesion, and Gensini score was the product of this multiplication.

Angiographic coronary thrombus burden was scored based on 5 grades, and the classification of thrombus burden is demonstrated in Table 1 in detail. We primarily calculated TIMI thrombus grade based on the initial diagnostic angiogram after restoring antegrade flow through guidewire or small balloon dilatation [15–17]. We then stratified the final TIMI thrombus grades as low thrombus burden or high thrombus burden, based on scores 1 to 3 or 4 to 5, respectively.

### 2.3. Statistical Analysis

The analysis of the results was performed using the Statistical Package for Social Sciences (SPSS, Chicago, Illinois, USA), version 16.0 software for Windows. Data were tested for normal distribution using the Kolmogorov–Smirnov test. The categorical variables were shown as numbers of cases with percentages, and the normally distributed continuous variables were shown as mean ± standard deviation. Median values (minimum-maximum) were used for the continuous variables which were not normally distributed. Student's *t*-test was used for the analysis of the continuous variables which were normally distributed, and the *χ*^2^ test was used for the categorical variables. A *p* value of 0.05 was considered statistically significant. Pearson and Spearman correlation coefficients were used for correlation analysis where appropriate.

## 3. Results

The baseline characteristics of the patients are summarized in Table 2. Serum chemerin and CRP levels were significantly increased in patients with STEMI. Besides, Gensini score was higher in STEMI patients among the angiographic parameters.

In STEMI patients with high thrombus burden, serum chemerin levels were significantly higher than the patients with low thrombus burden (581.5 ± 173.7 versus 451.3 ± 101.2 mg/dL, *p* < 0.001). CRP levels and peak CK-MB levels were higher, and LVEF and post-PCI TIMI flow were lower in patients with high thrombus burden (Table 3). Multivariate analysis was performed in these patients, and chemerin levels were still found to be significantly increased in patients with high thrombus grade (OR: 1.009 (1.005–1.014), *p* < 0.001).

When correlation analysis was performed, serum chemerin levels were found to be significantly correlated with CRP levels (*r*=0.47, *p* < 0.001) and peak CK-MB levels (*r*=0.376, *p* < 0.001). There was no correlation between the serum chemerin levels and Gensini score (*r*=0.083, *p*=0.355).

## 4. Discussion

In this study, the main finding was the significant relationship between circulating chemerin levels and the thrombus burden in patients with STEMI undergoing primary PCI. Besides, increased serum chemerin levels were also associated with increased jeopardized myocardial area which was assessed by peak CK-MB levels and increased level of inflammation determined by CRP levels. To the best of our knowledge, this is the first study in the literature evaluating the relationship between chemerin levels and thrombus burden in STEMI.

Inflammation and lipid hemostasis have been demonstrated to have a pivotal role in the pathogenesis of atherosclerotic coronary artery disease and acute coronary syndromes. The adipokines which are released from the adipose tissue such as chemerin, interleukin 6 (IL-6), leptin, and tumor necrosis factor- (TNF-) *α* play different roles in the inflammatory process causing development and acceleration of atherosclerosis and contributing to the presence of acute coronary syndromes [18–23]. It has been also found that serum chemerin levels are correlated with both arterial stiffness and coronary arterial plaques [11, 13, 24]. Lehrke et al. found a weak relationship about the relation between chemerin and coronary plaque burden and the number of noncalcified plaques in subjects undergoing CT angiography [11]. However, in some studies, serum chemerin levels have not been demonstrated to be related with the atherosclerotic plaque burden [25]. Herein, we could not find a significant relationship between the serum chemerin levels and extent of coronary artery disease; however, serum chemerin levels were significantly correlated with inflammatory markers and coronary thrombus burden.

In our study, one of the important findings was the relationship between serum CRP and chemerin levels. This finding may be a clue that chemerin is an important factor in the inflammatory process of acute STEMI. Increased CRP levels as a marker of inflammation have been shown to be related with increased morbidity and mortality in STEMI patients [26]. The relationship between high thrombus burden and CRP levels which has been recently shown by Niccoli et al. [27] is one of the explanations of this relationship. Herein, we have also found increased CRP levels in patients with thrombus burden. Besides, serum chemerin levels were also increased in these patients, and after multivariate analysis, this finding still persisted.

In our study, we have also found that higher serum chemerin levels were associated with higher peak serum CK-MB levels as a marker of increased necrotized myocardial tissue. Increased thrombus burden in patients with high chemerin levels may explain this finding, because in these patients, post-PCI TIMI frame counts were higher showing poorer perfusion of the jeopardized myocardial tissue possibly due to distal embolization of the increased thrombus burden.

In the pathogenesis of coronary artery disease and acute coronary syndromes, endothelial dysfunction is another important factor precipitating the development of atherosclerosis. The endothelium is the major regulator of vascular homeostasis and maintains the balance between vasodilatation and vasoconstriction, stimulation and inhibition of smooth muscle proliferation and migration, and thrombogenesis and fibrinolysis [28]. As we know, chemerin induces insulin resistance which is associated with endothelial dysfunction and inflammation in primary human skeletal muscle cells [29]. It was also shown that insulin resistance is associated with endothelial dysfunction in both peripheral arteries and coronary arteries [29]. Therefore, we can say that increased chemerin levels may cause more endothelial dysfunction resulting in increased thrombus formation and worse coronary arterial flow assessed by TIMI frame count in our study.

### 4.1. Study Limitations

The present study was cross sectional and collected data at one point in time, thus not allowing for inferences or changes over time. Another limitation of our study was the low number of patients. No significant clinical event developed in the hospitalization period of the patients, and therefore we could not assess the clinical significance of this newly discovered molecule. Besides, long-term follow-up of the patients were missing, because inclusion of patients has recently ended. A molecule which may predict the long-term clinical manifestations of acute STEMI would have been more valuable.

The strengths of our study were as follows: (a) a recently discovered molecule, chemerin, has been studied in a selected population, that is, STEMI patients undergoing primary PCI, and (b) plasma chemerin levels were firstly discovered to be related to the coronary thrombus burden in the setting of STEMI which might lead further studies and new treatment strategies targeting chemerin.

## 5. Conclusion

Results from our study have demonstrated for the first time that chemerin levels were higher in STEMI patients with greater thrombus burden and higher level of inflammation. The main role of chemerin in the pathogenesis of acute STEMI should be investigated by further studies.

## Figures and Tables

**Table 1:** The TIMI thrombus burden classification^a^.

Grade	Definition
Grade 0	No angiographic characteristics of thrombus are present.
Grade 1	Possible thrombus is present with such angiographic features (decreased contrast density, haziness of contrast, irregular lesion contour, a smooth convex meniscus at the site of a total occlusion, suggestive, but not firmly diagnostic of thrombus).
Grade 2	Definite thrombus is present with greatest dimension of thrombus (<1/2 vessel diameter).
Grade 3	Definite thrombus appears in multiple angiographic views (greatest dimension from >1/2 to <2 vessel diameters).
Grade 4	Definite large size thrombus is present (greatest dimension > 2 vessel diameters).
Grade 5	Definite complete thrombotic occlusion of a vessel (a convex margin that stains with contrast, persisting for several cardiac cycles); angiographic detection of a grade 5 TIMI thrombus leads to further exploration of the occlusive thrombotic content. Either a PCI guidewire or a small balloon is advanced across the thrombotic total occlusion. Crossing the thrombus results in restoration of antegrade flow in the treated vessel. Consequently, the ensuing coronary angiogram enables restratification of the underlying residual thrombus (final TIMI thrombus grade).

^a^Data taken from [15–17].

**Table 2:** Baseline characteristics of the patients.

	STEMI patients (*n*=97)	SAP patients (*n*=30)	*p* value
Age (years)	59.1 ± 7.4	59.3 ± 8.3	0.891
Sex (male)	62 (63.3%)	20 (66.7%)	0.734
Hypertension	67 (68.4%)	21 (70.0%)	0.866
Diabetes mellitus	20 (20.4%)	7 (23.3%)	0.731
Hyperlipidemia	42 (42.9%)	15 (50.0%)	0.491
Smoking	51 (52.0%)	14 (46.7%)	0.606
LVEF (%)	51.9 ± 8.1	59.9 ± 8.8	<0.001
Serum creatinine (mg/dL)	0.9 ± 0.2	0.9 ± 0.2	0.376
CRP (mg/L)	11.0 ± 5.2	6.3 ± 3.4	<0.001
Serum chemerin level (ng/ml)	521.0 ± 157.2	268.3 ± 86.4	<0.001
Gensini score	59.1 ± 15.1	51.0 ± 14.3	0.011

**Table 3:** The characteristics of STEMI patients according to their thrombus burden.

	Low thrombus burden (grade 0–3) *n*=45	High thrombus burden (grade 4–5) *n*=52	*p* value
Age (years)	58.9 ± 7.3	59.7 ± 9.1	0.641
Sex (male)	28 (%62.2)	33 (%63.5)	0. 900
Hypertension	28 (%62.2)	38 (%73.1)	0.253
Diabetes mellitus	8 (%17.8)	12 (%23.1)	0.520
Hyperlipidemia	18 (%40.0)	23 (%44.2)	0.674
Smoking	24 (%53.3)	27 (%51.9)	0.890
LVEF (%)	53.5 ± 7.7	50.4 ± 8.2	0.066
Serum creatinine (mg/dL)	0.9 ± 0.4	0.9 ± 0.4	0.392
Peak CK-MB level (IU/L)	111.2 ± 67.1	141.4 ± 79.4	0.045
CRP (mg/L)	9.8 ± 4.1	12.2 ± 5.8	0.022
Serum chemerin level (ng/ml)	451.3 ± 101.2	581.5 ± 173.7	<0.001

*Angiographic characteristics*			
Culprit lesion			
LAD	9 (20.0%)	12 (23.1%)	
Cx	16 (35.6%)	21 (40.4%)	0.731
RCA	20 (44.4%)	19 (36.5%)	
TIMI thrombus grade	2.3 ± 0.8	4.4 ± 0.5	<0.001
TIMI flow before primary PCI	0.3 ± 0.7	0.3 ± 0.6	0.401
TIMI flow after primary PCI	2.7 ± 0.5	2.4 ± 0.7	0.022
Post-PCI TIMI frame count	31.3 ± 10.8	37.1 ± 14.6	0.031
Gensini score	58.3 ± 14.5	60.1 ± 15.7	0.549
